# The salutary action of vitamin E on reproductive performance and renal functions in cadmium-exposed male mice

**DOI:** 10.5455/javar.2024.k857

**Published:** 2024-12-29

**Authors:** Mahabub Alam, Afrina Mustari, Samia Rashid, Shaima Alam Shimu, Tazmim Akter, Airin Akter, Mohammad Alam Miah, Emdadul Hauqe Chowdhury

**Affiliations:** 1 Department of Physiology, Faculty of Veterinary Science, Bangladesh Agricultural University, Mymensingh, Bangladesh; 2 Department of Pathology, Faculty of Veterinary Science, Bangladesh Agricultural University, Mymensingh, Bangladesh

**Keywords:** Cadmium, Vitamin E, Reproduction, Kidneys, Histomorphology

## Abstract

**Objectives:**

The research is based on the assessment of the beneficial role of vitamin E (vit-E) supplementation on the reproductive and renal functions in Cadmium (Cd)-exposed male mice.

**Materials and Method:**

Mice (*n* = 15 in each group) were kept untreated (Group A) or fed with cadmium chloride (CdCl_2_) (3.5 mg/kg, Group B) per day or both CdCl_2_ (3.5 mg/kg) with vit-E supplementation (200 mg/kg, Group C) daily for 60 days. Mice were euthanized, blood samples were collected, and serum was prepared for biochemical and hormonal analysis. Sperm motility, sperm concentration, testis weight, and diameter were taken. Tissues from the kidneys and testicles were collected in 10% neutral buffered formalin for histotexture study.

**Results:**

Cd treatment reduced the serum thyroxine (T_4_) and testosterone levels, but vit-E supplementation increased both T_4_ and testosterone levels in the Cd-treated mice. Cd treatment decreased sperm motility and concentration, testicular weight, and diameter, and induced degenerative changes in the seminiferous tubules, which significantly improved upon vit-E supplementation. Increased serum urea, uric acid, and creatinine concentrations, along with cellular infiltration in the renal tubular epithelium and glomerular hyperplasia, were found in the Cd-treated mice, which were not found in the vit-E-supplemented mice.

**Conclusion:**

The study points to the harmful consequences of Cd on reproductive performance and renal functions that could potentially be mitigated upon vit-E supplementation in the diet.

## Introduction

Cadmium (Cd) exposure is especially alarming because of its bioaccumulative characteristics, which can result in increased concentrations along the food chain, ultimately impacting both wildlife and human populations [1]. The bioaccumulation of Cd is concerning due to its prolonged environmental persistence, exhibiting a half-life of roughly 25–30 years in biological systems [2]. Cd’s harmful effects arise from multiple mechanisms, including oxidative stress, disruption of cellular signaling pathways, and interference with normal physiological functions [3]. Cd hinders Sertoli cell multiplication and triggers deoxyribonucleic acid (DNA) damage, cell death, and abnormal ultrastructure [4]. Cd significantly reduces the motility, vitality, and acrosomal reaction of sperm, and these changes connect with alterations in the sperm-specific Ca^2+^ channel and the sperm-specific K+ channel [5]. In addition to being a carcinogen, Cd has the potential to operate as an endocrine disruptor [6]. A low dose of Cd induces testicular damage, as seen by lower serum testosterone levels [5]. Cd affects thyroid hormones such as thyroid-stimulating hormone, thyroid peroxidase antibody, and free thyroxine (T_4_), which are the most classic indicators of thyroid dysfunction, comprising hypothyroidism, hyperthyroidism, and autoimmune thyroid diseases [7]. Cd decreases the ability of rats to reproduce by causing substantial deterioration and harm to the testicles, impairing the seminiferous tubules, and resulting in necrosis [8]. Cd can also impede heme production, disturb the structure of cell membranes, and induce malfunction in lipid metabolism [9]. In addition, Cd may impact the functionality of renal epithelial cells [10]. The kidneys are also affected by Cd-induced toxicity, and the toxicity in the kidneys seems to be reliant on renal Cd concentrations during exposure [11].

An antioxidant is stored in the membranes of cells, notably mitochondrial membranes and microsomes, disrupting the function of free radicals, preventing the creation of lipid peroxides, and also shielding sperm from reactive oxygen species (ROS) disruption [12]. Clinical research has demonstrated that vitamin E (vit-E) is useful in the treatment of reproductive disorders [13]. Oxidative stress responses are caused by a lack of this vitamin in testicular tissue, which reduces testosterone synthesis and spermatogenesis, indicating that the process of spermatogenesis cannot be sustained without vit-E [14]. In a sleep-deprived Wistar rat, vit-E seems to have a stronger influence on testosterone levels [15]. Thyroid hormones are considerably enhanced by vit-E supplementation owing to their antioxidant properties [16]. Vit-E dramatically improves testicular morphologic characteristics [17]. Vit-E increases kidney weight and function [18]. Vit-E also functions as a potent antioxidant and improves the overall histology of the kidney in male rats [19]. When it comes to the impact that vit-E has on the function of the kidneys and testicles of Swiss albino mice treated with Cd, there is a significant lack of knowledge accessible. The current research investigated the impacts of vit-E on hormonal and testicular physiological parameters along with renal functions in Cd-intoxicated male albino mice.

## Materials and Methods

### Ethical statement

The Animal Care and Ethics Committee of Bangladesh Agricultural University (BAU), Mymensingh-2202, granted permission for the research investigation (Ref. No. AWEEC/BAU/2024 (30)).

### Mice and living circumstances

For the purpose of the study, 45 male Swiss albino mice, weighing between 25 and 27 gm and with an average age of 3.5 weeks, were enrolled. The Department of Physiology at BAU maintained mice in a standard housing environment with a 12-h light-dark cycle and a temperature of 26°C ± 2°C. The mice received food and water *ad libitum*.

### Experimental layout and sample collection

Three groups of fifteen mice per group were allocated by a random selection process. Group A (control) was supplied with regular mouse pellets. The mice in groups B and C were provided with Cd (3.5 mg/kg/day) in drinking water and both Cd (3.5 mg/kg) daily in drinking water with vit. E (200 mg/kg) in feed, correspondingly. The study was conducted for a duration of 60 days. Mice had been subjected to a whole night of fasting before sacrifice. Then the mice were transferred into an airtight container with cotton that had been presoaked in diethyl ether once at a time. The mice were observed for signs of unconsciousness after some time. A sterile syringe was used to draw a blood sample straight from the heart and place it into a tube devoid of anticoagulant to prepare serum. For hormonal and biochemical analysis, the serum was kept at –20°C after being cleared by centrifugation for 15 min at 3,000 rpm. The testes and kidneys from each mouse were collected in 10% neutral buffered formalin following total blood withdrawal using phosphate-buffered saline perfusion.

### Hormonal and biochemical assays

The levels of serum testosterone as well as serum T_4_ had been measured using a testosterone radioimmunoassay kit (Berthold, Germany) and a T_4_ radioimmunoassay kit (Berthold, Germany), correspondingly, at the Institute of Nuclear Medicine and Allied Sciences, Mymensingh Medical College, Mymensingh, Bangladesh, following established protocols. The levels of serum urea, uric acid, and creatinine were measured at Professor Dr. Mohammad Hussain Central Laboratory, BAU, Mymensingh. This was done by applying a radioimmunoassay kit (Berthold, Germany) and following the normal technique.

### Testicular and sperm physiology

The testes were immersed in physiological saline and then carefully transferred onto chromatographic sheets. A digital weighing machine was employed to weigh the specimens while the diameter of the testes was measured using slide calipers. Sperm motility and concentration were determined after dissecting the epididymis. A small piece of the epididymal tissue was warmed on the slide with a cover slip placed on it, and then the motility percentage was evaluated. As previously done in our work [20], epididymal sperm were collected and counted. Cauda epididymis of the mice was dissected out in a Petri dish and minced gently with scissors for this procedure. The tissue was then put in a 4 ml test tube with 2 ml of prewarmed normal saline at 37°C, and the sperm was allowed to release in 5-10 min. Last of all, the sperm concentration and motility were counted using a Neubauer chamber, and the samples were analyzed with the help of a microscope having a high-power lens.

### Histopathology

For processing, sectioning, and staining the well-fixed tissues; normal technique and processing was used by coordinating with the pathology department, BAU, following the standard protocol [21]. The stained slides were viewed using a photomicroscope (Model: CX43, Olympus Corporation, Tokyo, Japan) was used for imaging. Johnsen scores were used to evaluate spermatogenesis in seminiferous tubules [22]. At a magnification of 40×, 20 cross-sectional segments of seminiferous tubules were examined in every sample and ranked from 1 to 10. 10: Shows perfect tubules and complete spermatogenesis; 9: Multiples of sperm but poor spermatogenesis; 8: Few sperm; 7: No sperm but good number of spermatids; 6: Few spermatids; 5: Few spermatids but no sperm and good number spermatocytes; 4: Few spermatocytes; 3: Only spermatogonia present; 2: No germ cells 1: Neither germ cells nor Sertoli cells.

### Statistical analysis

Before analysis, all data was gathered and inputted into Microsoft Excel 2016. The data was then subjected to analysis using GraphPad Prism 5.0, specifically employing a one-way Analysis of Variance (ANOVA) with Bonferroni multiple comparison test. Significance levels were set at *p* ≤ 0.05, *p ≤ 0.01, and ***p ≤ 0.001.

## Results and Discussion

### Serum T_4_ and testosterone concentration

**Figure 1.**
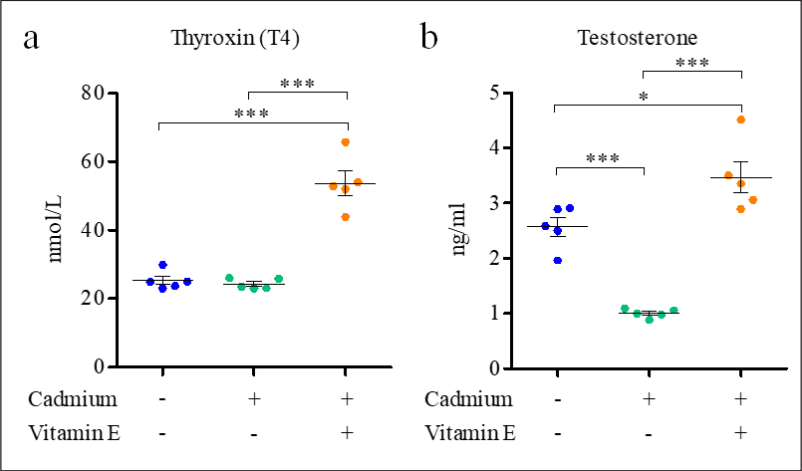
Impact of Cd and vit-E on T_4_ and testosterone of Male Swiss Albino mice. The mice were categorized into groups; control group; cadmium-group; Cd-vit-E group. In their serum, levels of T_4_ and testosterone were determined. The total data is expressed graphically as mean ± SEM which has been derived from five pooled groups each consisting of three mice. The numerical data was analyzed by one way ANOVA followed by Bonferroni test for multiple comparisons with *p ≤ 0.05 and ***p ≤ 0.001.

The hormonal assay disclosed that the serum T_4_ level (Fig. 1a) was comparable between control (25.30 ± 1.20 nmol/l) and Cd-treated (24.28 ± 0.67 nmol/l) mice, which increased considerably (*p < *0.001) in the group given vit-E supplements together with Cd (53.72 ± 3.50 nmol/l) (Fig. 1a). In contrast to the control group (2.56 ± 0.17 ng/ml), the serum testosterone level (Fig. 1b) in the Cd-treated group (1.00 ± 0.03 ng/ml) declined significantly (*p < *0.001) (Fig. 1b). However, similar to the T_4_ level, supplementation of vit-E in the Cd-administered mice significantly (*p < *0.05) enhanced the serum testosterone concentration (3.46 ± 0.28 ng/ml). Taken together, the blood levels of testosterone and T_4_ were higher when vit-E was supplemented. in mice treated with Cd, indicating a retrieval action of the vit-E in Cd-induced toxicity. The study validates earlier findings that low levels of testosterone and sex hormone-binding globulin are linked to blood Cd levels [20]. Cd exposure has been reported to harm the reproductive system of rodents, resulting in changes in sex hormone levels [23]. The vit-E supplementation has been found to significantly increase the testosterone levels in this study. Furthermore, vit-E boosts plasma levels of gonadal testosterone and increases fertilization potential [24]. Cd exposure lowers hepatic membrane-bound deiodinase activity via lipid peroxidation of the membrane as well as directly inhibiting T_4_ synthesis in the thyroid gland [25]. In our investigation, we found a rise in T_4_ concentration in vit-E-supplemented rats. Treatments using antioxidants such as vit-E could be beneficial in lowering hypothyroidism-induced oxidative damage by increasing T_4_ levels [26].

**Table 1.** Effect of Cd and vit-E on the weight and diameter of testis.

Group	Treatment	Testis weight (gm)		Testis diameter (cm)	
		Left testis Mean ± SEM	Right testis Mean ± SEM	Left testis Mean ± SEM	Right testis Mean ± SEM
A	Control	78.33 ± 1.66	75.67 ± 2.60	0.70 ± 0.03	0.67 ± 0.03
B	Cd	74.33 ± 2.33	72.67 ± 1.45	0.56 ± 0.03*	0.63 ± 0.03
C	Cd + vit-E	95.33 ± 7.51*	93.33 ± 9.95*	0.76 ± 0.01	0.70 ± 0.01

Note: Data indicate mean ± SEM. One-way ANOVA with Bonferroni multiple comparison test. Values with asterisks (*) superscripts are significantly different from control (*p* < 0.05).

**Figure 2.**
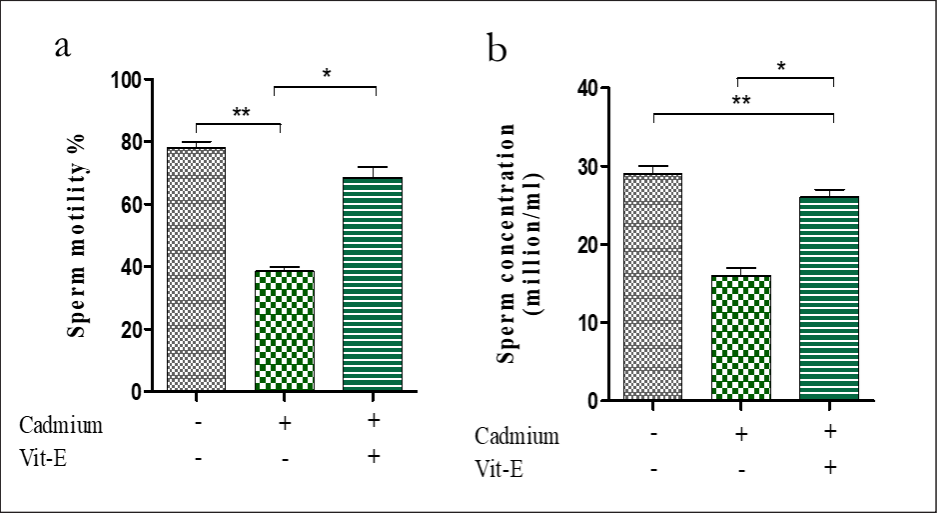
Impact of Cd and vit–E on sperm motility and concentration of Male Swiss Albino mice. The mice were categorized into groups; control group; Cd-group; Cd-vit-E group and sperm motility and concentration were analyzed. The total data is expressed graphically as mean ± SEM. The numerical data was analyzed by one way ANOVA followed by Bonferroni test for multiple comparisons with *p ≤ 0.05 and **p ≤ 0.01.

### Weight and diameter of testes

Next, the impact of Cd treatment and vit-E supplementation on the weight and diameter of testes (Table 1) of mice was evaluated. The weight of the left testis was comparable between the control (78.33 ± 1.66 gm) and Cd-treated mice (74.33 ± 2.33 gm). Whereas the weight of the left testis grew considerably (*p < *0.05) in the mice that were administered Cd and vit-E supplements (95.33 ± 7.51 gm), suggesting that vit-E has a positive impact on the gonadal structure of Cd-treated mice (Table 1). Similarly, the weight of the right testis was comparable between the control (75.67 ± 2.60 gm) and Cd-treated (72.67 ± 1.45 gm) mice; an increase in the weight was noticed in Cd-treated mice upon vit-E supplementation (93.33 ± 9.95 gm). On the other hand, the diameter of the left testis has decreased drastically (*p < *0.05) in Cd-administered (0.56 ± 0.03 cm) mice compared to the control group (0.70 ± 0.03 cm); supplementation of vit-E increased (*p > *0.05) the testis diameter in the Cd-treated mice. While the diameter of right testes was comparable between the control (0.67 ± 0.03 cm) and Cd-treated (0.63 ± 0.03 cm) mice, a slight increase (*p > *0.05) was noticed upon vit-E supplementation in the Cd-treated group (0.70 ± 0.01 cm). Collectively, Cd treatment reduced the weight and diameter of the testes of mice, which was rescued and further improved upon with vit-E supplementation. Testicular mass has a high and significant correlation with sperm generation, making it a crucial factor in the assessment of male reproductive capacity. Cd exposure significantly reduced testis diameter, while vit-E supplementation in Cd-exposed mice resulted in significant enhancement in testis weight and diameter [27]. The current results correlate with the results.

### Sperm motility and concentration

The sperm motility (Fig. 2a) was comparable between the control (78.0 ± 2.0%) and Cd-treated mice (38.50 ± 1.5%). Whereas it increased considerably (*p < *0.05) in the mice that were administered Cd and vit-E supplements (68.50 ± 3.5%), suggesting that vit-E has a positive impact on improving the sperm quality of Cd-treated mice. Similarly, the sperm concentration (Fig. 2b) in Cd-treated (16.0 ± 1.0 million/ml) mice was lower compared to the control group (29.0 ± 1.0 million/ml), where the reduction was significantly (*p < *0.01) restored by elevation of the sperm concentration in vit-E-supplemented Cd-treated mice (25.0 ± 1.0 million/ml). This work is consistent with Oliveria, wherein treated mice with Cd chloride (CdCl_2)_ showed a reduction in sperm concentration [28]. Cd produces oxidative stress [29], and ROS can impede sperm motility by peroxidizing membrane lipids and decreasing axonemal protein phosphorylation [30]. Vit-E improves sperm quality in sodium arsenite-treated rats [17]. The current results were also consistent with the observations made by Elayapillai [31], who discovered vit-E can enhance sperm health. Its ability to function as an antioxidant has shown its usefulness in mitigating the adverse consequences of exogenous and toxic substances [32].

**Figure 3.**
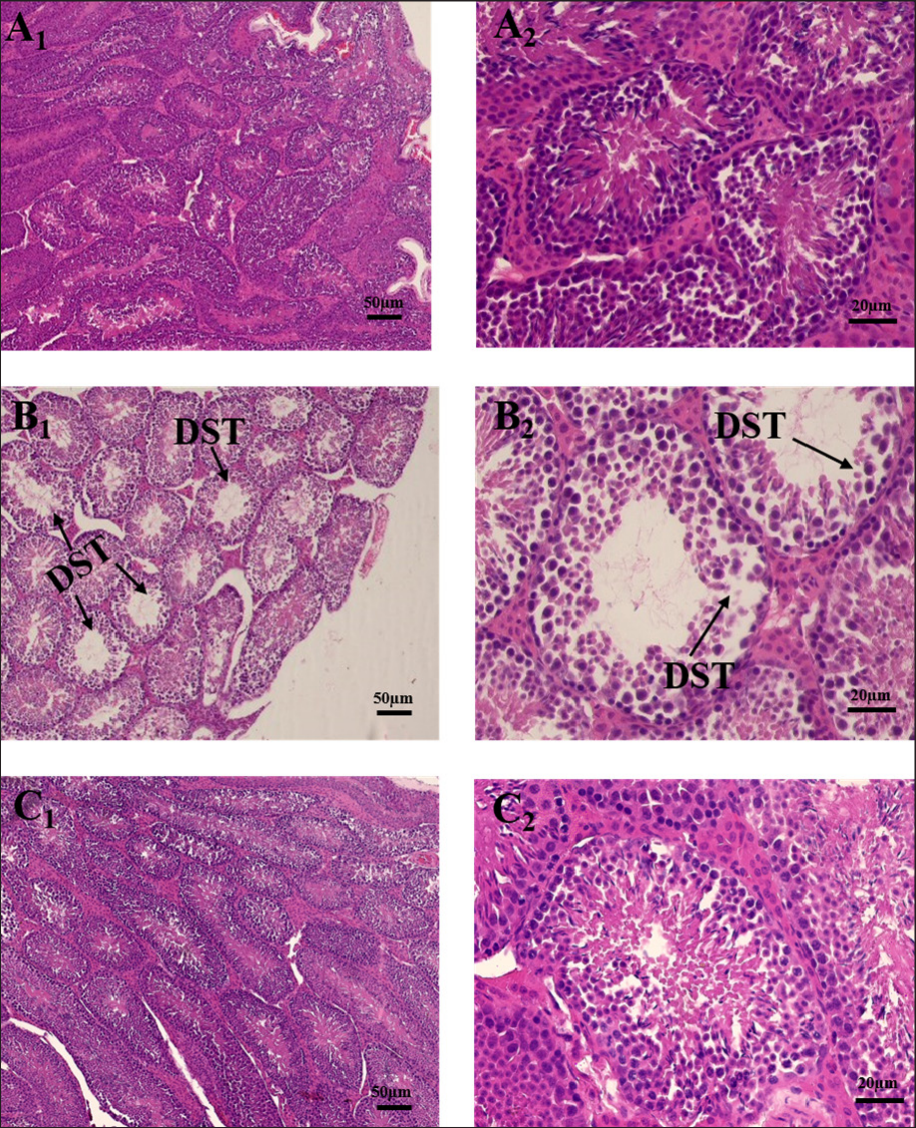
Impacts of Cd and vit-E on testis of mice. Photomicrograph of testis in mice of control (A_1_, A_2_), Cd treated group (B_1_, B_2_) and Cd + vit-E treated group (C_1_, C_2_) at 100× and 400× magnification respectively. DST = degenerative seminiferous tubule.

### Histoarchitecture of testes

**Figure 4.**
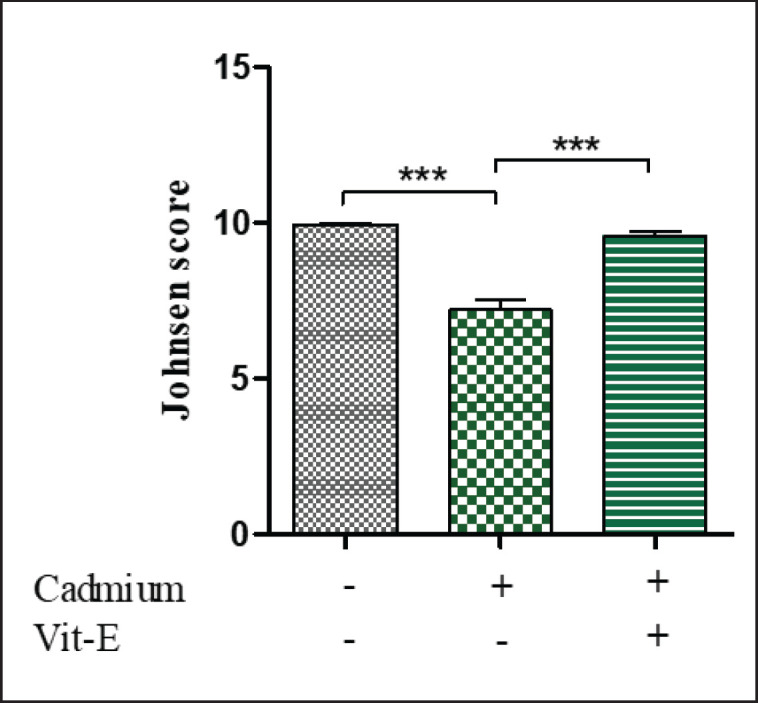
Impact of Cd and vit-E on Johnsen score (seminiferous tubule’s functionality) of Male Swiss Albino mice. The mice were categorized into groups; control group; Cd-group; Cd-vit-E group and Johnsen score were quantified. The total data is expressed graphically as mean ± SEM. The numerical data was analyzed by one way ANOVA followed by Bonferroni test for multiple comparisons with ***p ≤ 0.001.

The histopathological (Fig. 3) study revealed that Cd causes degeneration of seminiferous tubules. Whereas the degenerative changes were recovered after vit-E supplementation (Fig. 2). The Mean Johnson’s score (MJS) was applied for the evaluation of the functioning of the seminiferous tubules. To assess 20 cross-sectional portions of seminiferous tubules of every group, 40× magnification was applied (Fig. 4). Untreated mice provided MJS of 9.95 ± 0.04 for normally developed seminiferous tubules, while mice receiving Cd exhibited MJS of 7.2 ± 0.31 for damaged seminiferous tubules. The difference found in Cd+Vit E group was statistically significant with the other group (positive control group). The group supplemented with vit-E and Cd has increased the MJS to 9.58 ± 0.15, which could be observed in the impacts of vit-E on synthesizing the male reproductive system of Cd intoxication. These findings are in concordance with those of Ekhoye [33], who reported that Cd-altered seminiferous tubules in treated testes are damaged and degenerated. Cd has an adverse classic toxic effect of causing direct injury to seminiferous tubules, Sertoli cells, and the blood-testis barrier that in turn leads to reduced sperm production [34]. Cd also triggers a notable increase in the thickness of the seminiferous epithelium, along with cellular degradation and necrosis [35]. In agreement with previous studies on Cd’s negative effects on male reproductive health, Cd exposure causes extensive necrosis and vacuolization of seminiferous tubular cells with absence or impaired spermatogenesis, interstitial tissue edema, hemorrhages, and severely affected gross morphological structure, which decreased Johnsen’s score [36]. Vit-E functions as a powerful antioxidant, repairing testicular tissue, protecting DNA against oxidative stress, as well as preventing the production of free radicals [37].

### Renal function test

**Figure 5.**
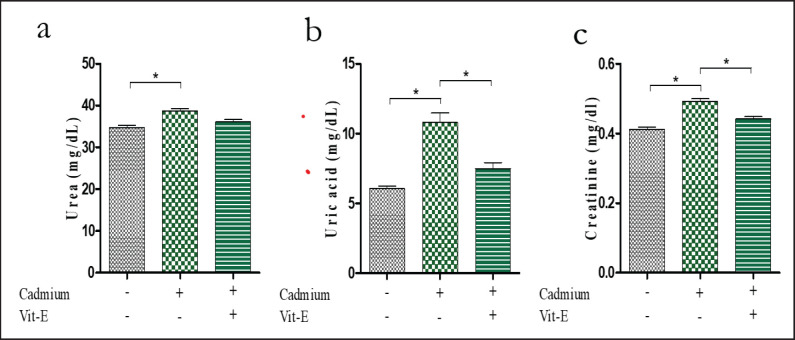
Impact of Cd and vit-E on renal biomarkers of Male Swiss Albino mice. The mice were categorized into groups; control group; Cd-group; Cd-vit-E group and serum urea, uric acid and creatinine level were analyzed. The total data is expressed graphically as mean ± SEM. The numerical data was analyzed by one way ANOVA followed by Bonferroni test for multiple comparisons with *p ≤ 0.05.

Then, we evaluated the effect of Cd treatment on the serum urea, uric acid, and creatinine levels. The serum urea level was 34.70 ± 0.57 mg/dl in the control group which increased significantly in the Cd-treated (38.74 ± 0.55 mg/dl) mice (Fig. 5a). Supplementation of vit-E reduced (36.11 ± 0.52 mg/dl) significantly (*p < *0.001) the concentration of serum urea among the Cd-administered mice. The serum uric acid and creatinine also increased significantly after Cd treatment, which reduced after vit-E supplementation. The high level of serum urea, uric acid, and creatinine in Cd-administered mice indicated kidney damage, which was significantly improved upon vit-E supplementation (Fig. 5b and c). Zhao et al. [38] also found similar results that Cd induces plasma creatinine level. According to reports, after 3–6 h of exposure, Cd causes kidney proximal tubular cells in rats to die from exposure [39]. Cd induces apoptosis in rat renal epithelial cells and reduces nuclear factor-kB activity, whereas vit-E supplementation significantly reduces serum creatinine levels [35,40].

### Histotexture of kidney

**Figure 6.**
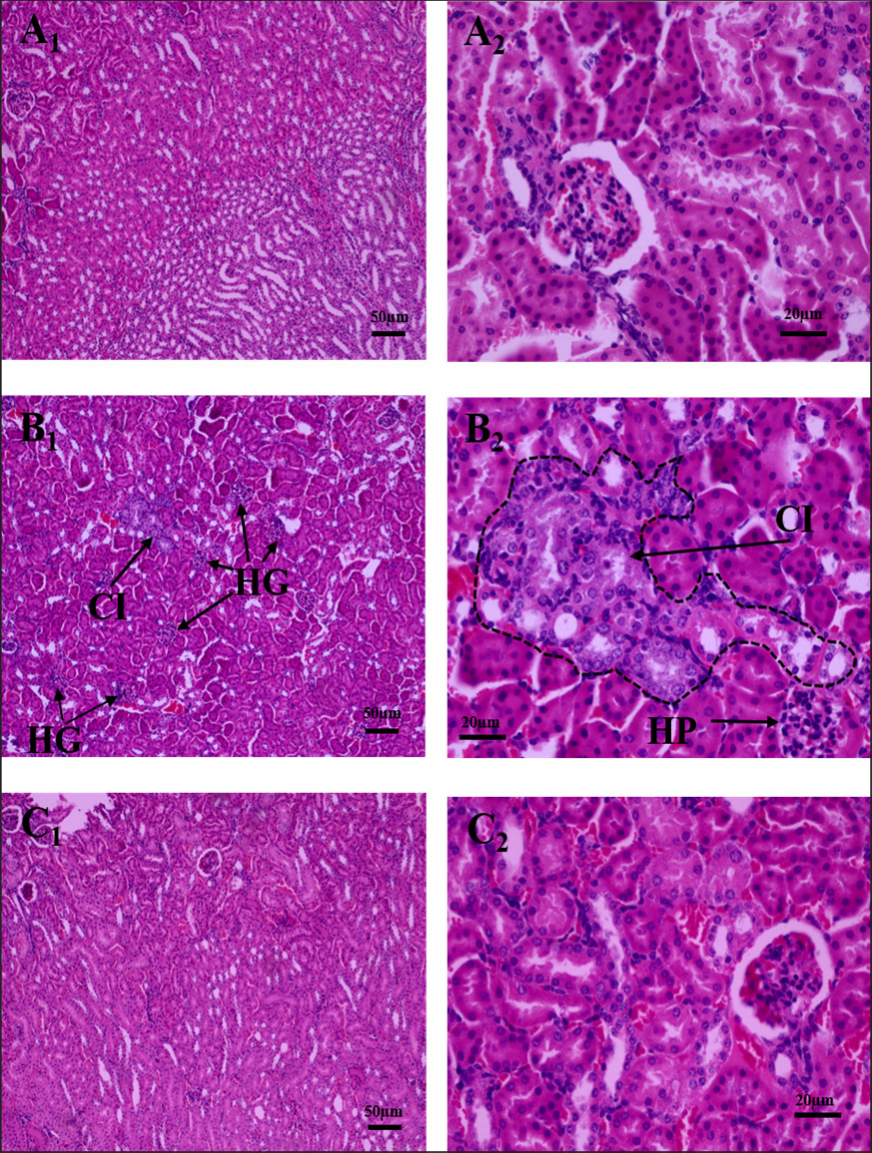
Effects of Cd and vit-E on histoarchitecture of kidney in mice. Photomicrograph of kidney in mice of control (A_1_, A_2_), Cd treated group (B_1_, B_2_) and Cd + vit-E treated group (C_1_, C_2_) at 100× and 400× magnification respectively. CI = cellular infiltration; HG = hyperplastic glomerulus.

The histoarchitecture of the kidney showed that there was cellular infiltration in the tubular epithelium with glomerular hyperplasia in Cd-treated mice. Almost all the alterations were minimized after vit-E supplementation (Fig. 6). Tubular damage was identified in the kidneys of Cd-intoxicated rats [41], as well as tubular damage and glomerular shrinkage in mice [42]. Cd-induced degeneration and hypertrophy of epithelial cells, as well as dilatation of the glomeruli, were seen in the kidney tubules [43,44]. The preventive action of vit-E supplementation was evidenced by better tubular function, diminished glomerulosclerosis and tubulointerstitial scarring, and reduced kidney and glomerular hypertrophy [45].

## Conclusion

The experimental results show that Cd toxicities in mice altered the various parameters associated with kidney and testicular damage. While supplementation of vit-E shows ameliorated effects against Cd-induced detrimental effects. To precisely understand the mechanism of how vit-E actually mitigates the negative impacts of Cd, more research is necessary.

## List of abbreviations

BAU, Bangladesh Agricultural University; Cd, cadmium; CdCl_2_, cadmium chloride; DNA, deoxyribonucleic acid; MJS, Mean Johnson’s score; ROS, reactive oxygen species; T_4_, thyroxine; vit-E, vitamin-E.
